# Impact of Resistant Starch on Body Fat Patterning and Central Appetite Regulation

**DOI:** 10.1371/journal.pone.0001309

**Published:** 2007-12-12

**Authors:** Po-Wah So, Wei-Sheng Yu, Yu-Ting Kuo, Clive Wasserfall, Anthony P. Goldstone, Jimmy D. Bell, Gary Frost

**Affiliations:** 1 Biological Imaging Centre, Imaging Sciences Department, MRC Clinical Sciences Centre, Imperial College, Hammersmith Hospital Campus, London, United Kingdom; 2 Molecular Imaging Group, Imaging Sciences Department, MRC Clinical Sciences Centre, Imperial College, Hammersmith Hospital Campus, London, United Kingdom; 3 Department of Nutrition, Dietetics and Food Science, School of Biomedical and Molecular Science, University of Surrey, Guildford, United Kingdom; 4 Department of Medical Imaging, Kaohsiung Medical University Hospital, Faculty of Medicine, College of Medicine, Kaohsiung Medical University, Kaohsiung, Taiwan; 5 Department of Radiology, Kaohsiung Medical University Hospital, Faculty of Medicine, College of Medicine, Kaohsiung Medical University, Kaohsiung, Taiwan; 6 Department of Pathology, University of Florida College of Medicine, Gainesville, Florida, United States of America; AgroParisTech, France

## Abstract

**Background:**

Adipose tissue patterning has a major influence on the risk of developing chronic disease. Environmental influences on both body fat patterning and appetite regulation are not fully understood. This study was performed to investigate the impact of resistant starch (RS) on adipose tissue deposition and central regulation of appetite in mice.

**Methodology and Principle Findings:**

Forty mice were randomised to a diet supplemented with either the high resistant starch (HRS), or the readily digestible starch (LRS). Using ^1^H magnetic resonance (MR) methods, whole body adiposity, intrahepatocellular lipids (IHCL) and intramyocellular lipids (IMCL) were measured. Manganese-enhanced MRI (MEMRI) was used to investigate neuronal activity in hypothalamic regions involved in appetite control when fed ad libitum. At the end of the interventional period, adipocytes were isolated from epididymal adipose tissue and fasting plasma collected for hormonal and adipokine measurement. Mice on the HRS and LRS diet had similar body weights although total body adiposity, subcutaneous and visceral fat, IHCL, plasma leptin, plasma adiponectin plasma insulin/glucose ratios was significantly greater in the latter group. Adipocytes isolated from the LRS group were significantly larger and had lower insulin-stimulated glucose uptake. MEMRI data obtained from the ventromedial and paraventricular hypothalamic nuclei suggests a satiating effect of the HRS diet despite a lower energy intake.

**Conclusion and Significance:**

Dietary RS significantly impacts on adipose tissue patterning, adipocyte morphology and metabolism, glucose and insulin metabolism, as well as affecting appetite regulation, supported by changes in neuronal activity in hypothalamic appetite regulation centres which are suggestive of satiation.

## Introduction

The link between obesity, adipose distribution, premature mortality and morbidity is one of the most enduring observations in the field of nutrition [1]. The rise in obesity and associated risks have become a major public health issue globally [2]. Although weight gain is ultimately the result of an overall positive energy balance, the environmental and genetic interplay that accounts for the dramatic rise in obesity is not fully understood. Nutritionally, there have been many significant changes in the profile of energetic nutrients over the last 100 years [3]. By far the largest research focus has been towards the impact of dietary fat on body composition. This has ignored not only the decrease in total carbohydrate intake but a change in the quality of carbohydrate that we have consumed over the last century. Recently, interest has been stimulated in understanding the relationship between dietary carbohydrate, appetite regulation, body weight and body composition [4], [5]. There is epidemiological evidence supporting changes in carbohydrate consumption have contributed to an increase in obesity [6]. Also, there have been reports of body composition being affected by carbohydrate type in small animals [7]. It would appear that slowly absorbed fermentable carbohydrate results in lower body fat content with no change in overall body weight [8]. At the present time, the mechanism for these observations is unclear. There is also some provisional evidence that diets high in resistance starch (RS) may have an effect on adipocyte metabolism by affecting the release of adiponectin [9]. The metabolic impact of RS in humans has recently been demonstrated by Robertson etal [10]. In this study supplementation with RS lead to a increase insulin sensitivity and a reduction in glycerol and free fatty acids across subcutaneous adipose tissue [10]. This is suggestive of a change in adipocyte metabolism that may lead to a change in insulin sensitivity. Also, fermentable carbohydrate such as RS and inulin has been demonstrated to increase the release of gut hormones with roles in appetite regulation and possibly, leptin release [9], [11].

Some of the effects of high intakes of RS and fermentable carbohydrate described above such as changes in body composition are suggestive of a central role in energy homeostasis. This has been technically difficult to evaluate. In this study, we use magnetic resonance imaging (MRI) and magnetic resonance spectroscopy (MRS) to determine body composition, not only in levels of traditional adipose fat but also ectopic fat levels such as intrahepatocyte and intramyocyte fat [12]. We also used a newly developed functional imaging method, manganese-enhanced MRI (MEMRI), to assess the impact of dietary starches on appetite centres in the hypothalamus [13], [14], [15]. This novel method allows in vivo assessment of brain activity, making it ideal for longitudinal dietary studies. Moreover, we have shown that neuronal activity is unaffected by the presence of Mn^2+^ ions in our protocol and that this technique produces similar results to traditional c-Fos expression based methods to detect neuronal activation [14].

We hypothesised that mice fed a diet high in RS as a model of fermentable dietary fibre would have similar body weights compared to those with a low RS intake but have differences in body composition, metabolic profile and central appetite regulation.

## Results

### Body Weight, Food Intake and Body Composition Data

**Figure 1:**
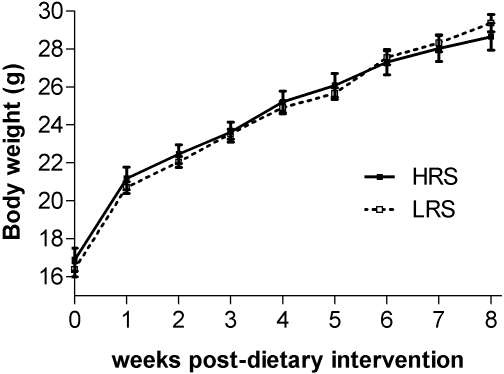
The effect of HRS and LRS diets on average body weights over the 8-week dietary interventional period. No significant difference was observed in the body weight between the two groups of animals [two way ANOVA with post-testing by Bonferonni correction, n = 16 and 20 for HRS and LRS groups, respectively.)

**Figure 2:**
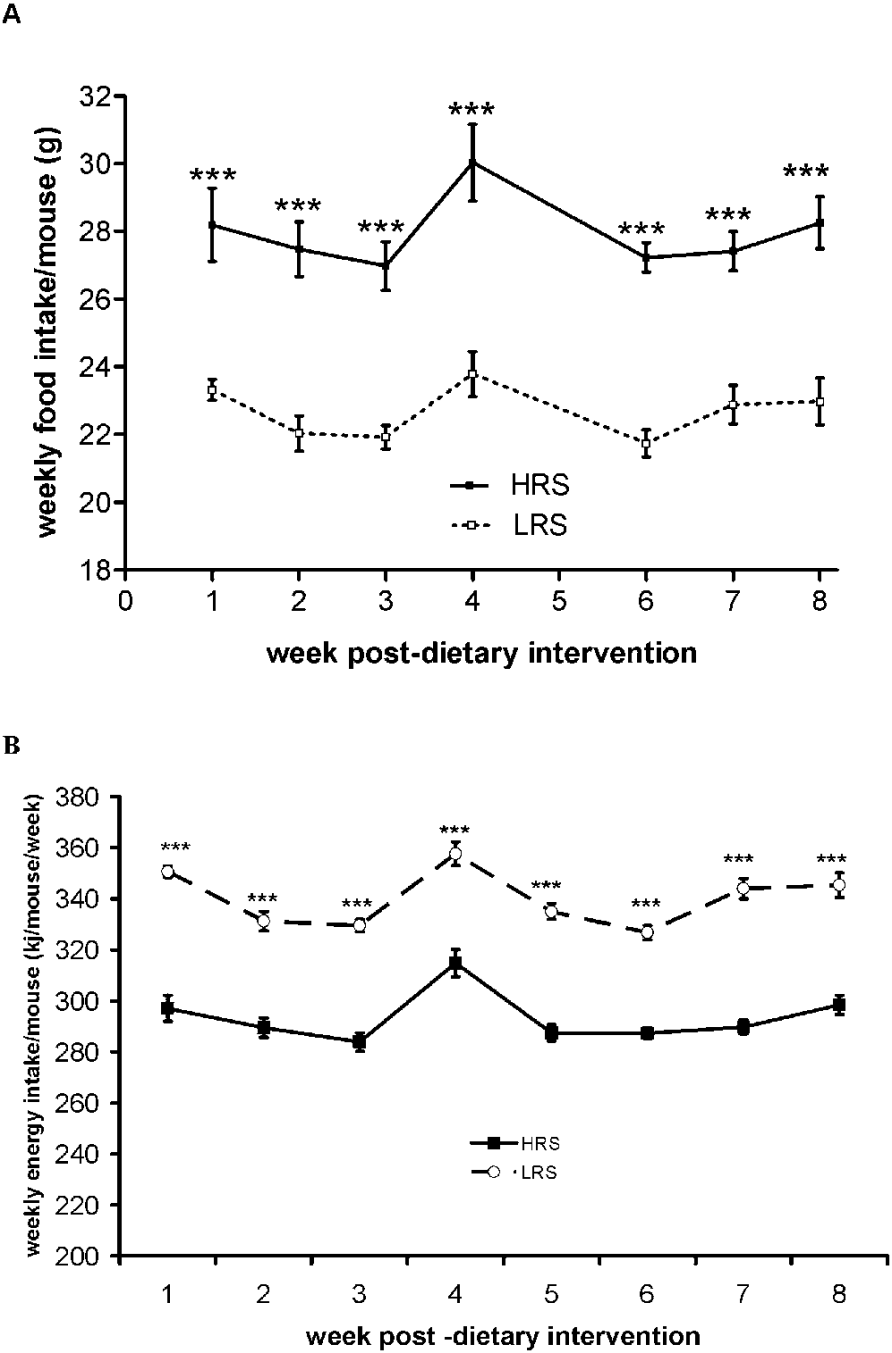
The effect of HRS and LRS on average weekly food and energy intake per mouse over the 8 week dietary intervention period. Figure 2a: The effect of HRS and LRS on average weekly food intake per mouse over the 8 week dietary interventional period. Figure 2b: The effect of HRS and LRS on average weekly energy intake (KJ/mouse/week) over the 8 week dietary intervention period. [***, significance at level P<0.001, two way ANOVA with post-testing by Bonferonni correction, n = 16 and 20 for HRS and LRS groups, respectively.]

**Figure 3:**
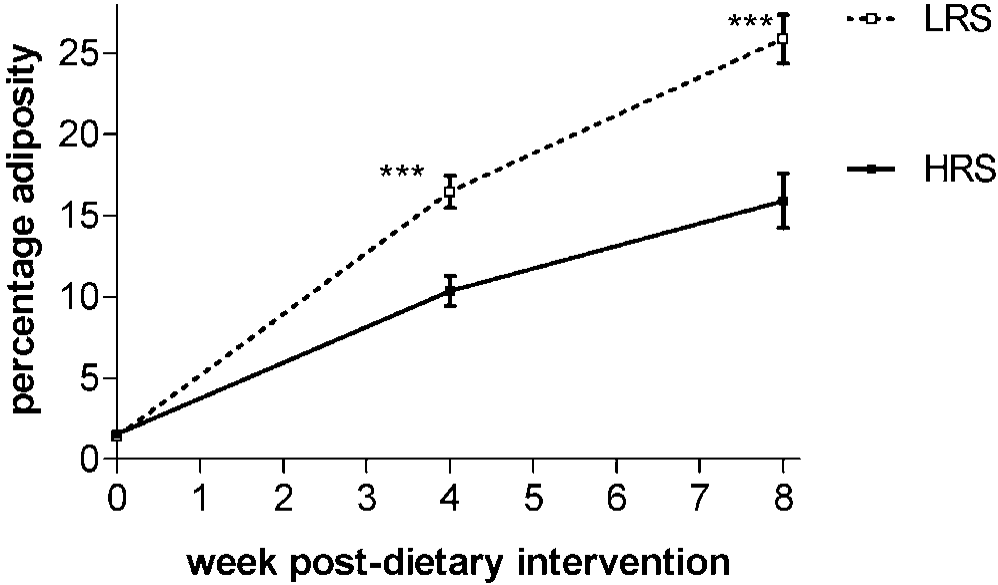
The effect of HRS and LRS diets on percentage adiposity over the 8 week dietary interventional period. [***, significance at level P<0.001, two way ANOVA with post-testing by Bonferonni correction, n = 16 and 20 for HRS and LRS groups, respectively.]

**Figure 4:**
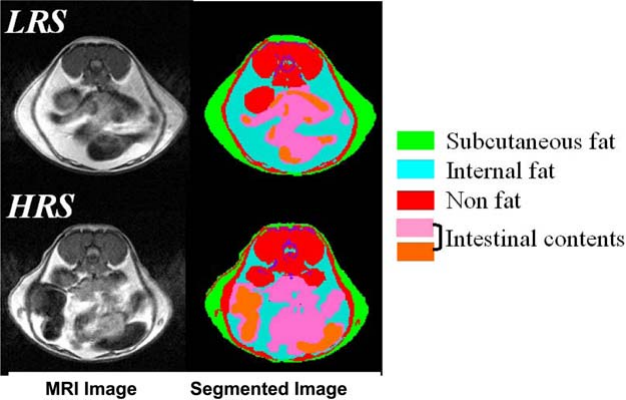
Typical transverse abdominal MRI and associated segmented images of mice on HRS and LRS diets.

Both groups of mice maintained for 8 weeks on the LRS and HRS diets had similar body weights throughout the time-course (Fig. 1). This was despite a significantly higher food intake by the HRS group (Fig. 2a), reflecting animal adaptation to the energy dilution effect of the RS enriched feed. However, the increase in total weight of food consumed by individual mice in the HRS group did not fully compensate for the more energy dilute feed, this includes estimation of the energy recovery from the high resistant starch feed through fermentation. In the high HRS the total energy intake remains significantly lower in the HRS mice (Fig 2b). Assessment of total percentage adiposity by whole body ^1^H MRS, indicated that despite similar weight gains between the two groups, the weight gained by the LRS group arise from deposition of adipose fat rather than lean tissue mass (Fig. 3). By segmentation analysis of whole body MRI data, visceral and subcutaneous adipose tissue deposits were determined (Fig. 4) to be higher in the LRS group (P<0.05). Visceral adipose tissue deposits were 2.45±0.27 g and 1.64±0.19 g for LRS and HRS groups (P<0.05), respectively, and subcutaneous adipose tissue, 3.74±0.37 g and 2.67±0.32 g for the LRS and HRS groups (P<0.05), respectively.

Greater deposition of lipids in hepatocytes were detected in the LRS group, relative intrahepatocellular lipid (IHCL) levels were 12.4±1.7 and 4.3±0.6 for the LRS and HRS groups, respectively (P<0.01). No difference in the levels of relative intramyocellular lipid (IMCL) were detected between the two dietary groups.

### Neuronal MEMRI

**Figure 5:**
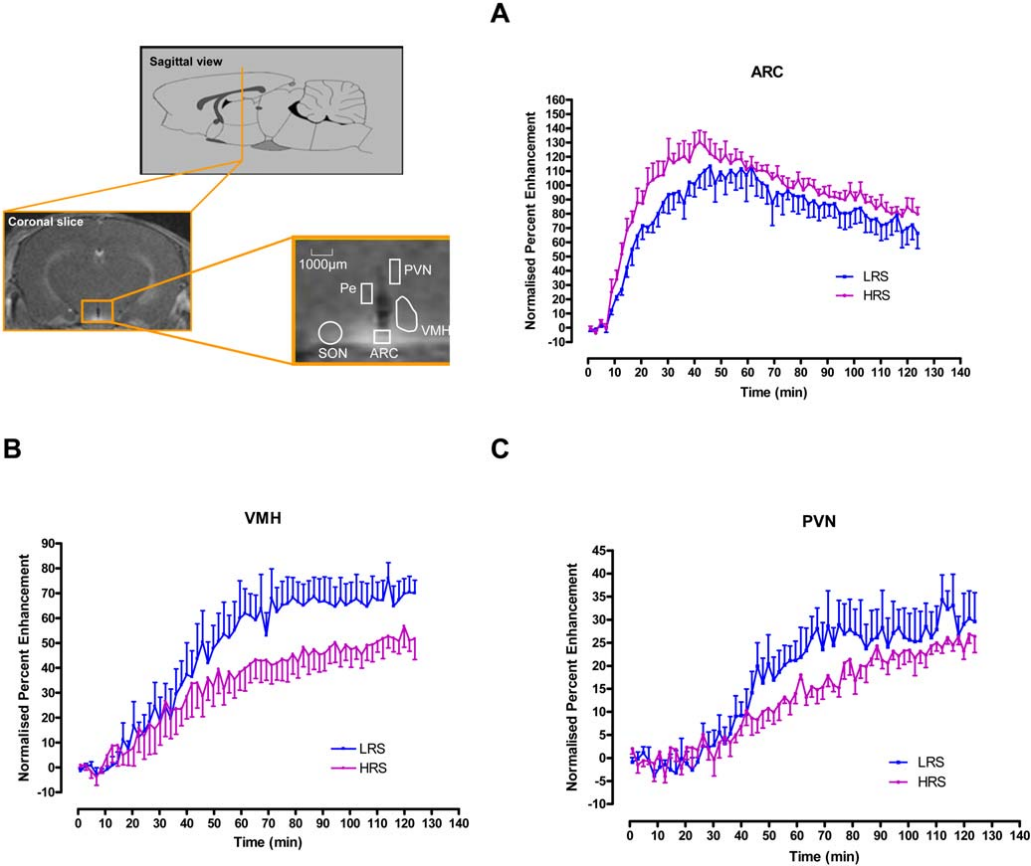
Representative baseline (pre-contrast) MRI images of the mouse brain showing assignment of regions of interest (ROIs) in various brain areas from which signal intensities (SI) were obtained. Time course of changes in SI (as a percentage of baseline) before and at various times after IV manganese chloride infusion in the (A) ARC, (B) VMH and the (C) PVN. Data are presented as means of three consecutive image acquisitions±SEM. Significant differences were observed in the VMH, and PVN brain areas (P<0.05) but not in the ARC. *Key:* ARC, arcuate hypothalamic nucleus; VMH, ventromedial hypothalamic nucleus; PVN, paraventricular hypothalamic nucleus; LRS, low resistant starch diet; HRS, high resistant starch diet.

Changes in signal intensity due to Mn^2+^ uptake was measured in selected regions of interest (ROI) on the MRI image (Figure 5). Figure 5 shows the time course for Mn^2+^ uptake as a percentage of baseline following IV infusion of Mn^2+^ in the arcuate hypothalamic nucleus (ARC), ventromedial hypothalamic nucleus (VMH) and paraventricular hypothalamic nucleus (PVN). The 4^th^ ventricle is located outside the blood-brain-barrier (BBB) and signal enhancement in this area allows for normalisation of the enhancement curves for Mn^2+ ^arrival time into the brain. No significant difference was observed in MEMRI of the 4^th^ ventricle between the LRS and HRS groups, suggesting Mn^2+^ uptake into this area outside the BBB to be the same in both groups. Although MEMRI in the ARC is greater in the HRS group compared to the LRS group, statistical difference was not reached (P<0.2). However, both the VMH and the PVN show significantly greater uptake in the LRS compared to the HRS group (P<0.05), indicating greater activation in the former dietary group.

### Adipocyte Data

Adipocytes isolated from the epididymal fat of LRS mice were found to be of a significantly larger size (7.89±0.12 µm) than those from the HRS mice (6.91±0.09 µm, P<0.001). A higher borderline insulin-stimulated glucose uptake was observed in the isolated adipocytes from the HRS group (273.0±50.7 % above baseline adipose tissue) compared to those from the LRS group (159.8±22.8 % above baseline adipose tissue, P<0.05).

### Plasma Analysis

Table 1 shows that plasma leptin and adiponectin were significantly higher in the LRS group compared to the HRS, despite no overall change in body weight. The glucose:insulin ratio, in the face of normal glycaemia was significantly higher in the HRS group suggesting ingestion of higher amounts of RS led to a more insulin-sensitive animal.

**Table 1:** The effect of resistant starch on plasma levels of insulin, glucose and selected adipokines.

Variable	High Resistant Starch		Low Resitant Starch		p
	Mean	SEM	Mean	SEM	
IL-6 (pmol/l)	1030.99	164.30	993.57	94.12	ns
Insulin (pmol/l)	75.25	44.33	106.23	48.89	ns
Glucose (mmol/l)	17.40	4.80	16.89	5.79	ns
I:G ratio	4.33	1.66	6.29	2.78	0.04
Leptin (pmol/l)	262.86	248.34	742.16	480.23	0.01
MCP-1 (pmol/l)	113.53	43.12	143.83	77.05	ns
Resistin (pmol/l)	81.74	31.05	103.56	55.48	ns
tPAI-1 (pmol/l)	26.87	15.72	41.63	15.95	0.06
Adiponectin (ug/ml)	14.83	4.97	22.51	9.25	0.01

IL-6 = Interleukin-6, MCP-1 = monocyte chemotactic protein-1, tPAI-1 = plasminogen activator inhibitor 1. Adiponectin is measured in ug/ml due to the different protein population.

## Discussion

Maintenance for 8 weeks on either the LRS or HRS diets lead to similar weight gain but differences in whole body adiposity. This is despite the HRS group consuming significantly more total weight of food in response to the greater energy dilution in the HRS feed. However despite this increase in total weight of food the energy intake in the animals on the HRS diet was significantly lower than the LRS group. For the animals in both groups to have the same total body weight would suggest the HRS animal had reduced energy expenditure, although this was not directly measured in this study. It is known that when animals are pair fed RS enriched feeds [7] there is no change in total body weight but similar changes in body composition as reported in this paper. However, it is difficult to unpick if some of the observations made in the study are due to a reduced energy intake and the metabolic compensation the animal uses to keep its body weight the same as the LRS group, but the vector for the changes is the HRS diet. These observations make the functionalMRI finding, discussed below, even more surprising.

Significant differences in total, subcutaneous and visceral adipose tissue was observed, the ingestion of a low RS diet leading to significantly greater deposition of adipose tissue. These observations are similar to those of Pawlak et al [7] in which rats maintained on a diet low in RS had significantly greater body fat and less lean body mass than those given a high RS diet, despite having similar mean body weights. We have not only confirmed the findings of others [7] but have added to such observations with the demonstration that the IHCL level is significantly higher in the animals fed a LRS diet. This may be a critically important observation given the mounting evidence to the central role of liver triacylglycerol in the pathogenesis of insulin resistance, a mechanism which is not really fully understood [16], [17]. It is interesting that in this study there is a increase in the insulin:glucose ratio in the LRS group, suggesting an increase in insulin resistance, a similar observation was made by Pawlak *et al*, in which a more comprehensive assessment of insulin and glucose metabolism was made [7]. This would suggest the change in body composition inducted by the HRS diet (lower percentage total body fat, arising from both lower levels of visceral and subcutaneous fat, and IHCL) has a positive impact on insulin and glucose handling.

We also observed changes in adipocyte morphology arising from differences in the diet, adipocytes were significantly larger in the LRS fed animals. There is a well established relationship between adipocyte size and insulin sensitivity, i.e., the larger the adipocytes, the lower their sensitivity to insulin [18]. This has been clearly demonstrated in weight loss regain experiments [19], [20]. This is consistent with the significantly lower insulin-stimulated glucose uptake rate observed in the adipocytes isolated from animals fed the LRS diet. Our team and others have demonstrated that a reduction in insulin-stimulated glucose uptake directly relates to a reduction in whole body insulin sensitivity [21], [22], [23]. The lower insulin-stimulated glucose uptake together with the significantly higher insulin:glucose ratio in the LRS group suggests these animals are moving towards a more insulin resistant state. Potential mechanisms for the changes in adipocyte size and insulin sensitivity are unclear. It is possible a link exists between short chain fatty acid production from the fermentation of RS and the change in adipocyte morphology. Recently, it has been reported that the G protein receptor, GPCR43, on adipocytes may play a role in adipocyte differentiation and proliferation [24]. The ligands for these receptors appear to be the short chain fatty acids, acetate and propionate, which although not measured in this study, has been well documented to be produced from fermentation of RS [25]. Further, there is recent evidence that activation of GPCR43 by acetate and propionate causes a decrease in leptin secretion and an increase in adipocyte differentiation [26]. The observations made in our study are similar to that by Slama *et al* who reported significantly larger adipocyte size and lower insulin-stimulated glucose oxidation in rats fed with diets of low fermentable content [27]. The same group also reported an increase in fatty acid storage enzymes and lipogenesis in such animals [28]. Taken together the results support the hypothesis that RS and possibly its fermentation in the large bowel may influence adipocyte metabolism.

An interesting observation is the significant difference in plasma leptin concentrations, with the LRS group having significantly higher levels despite similar body weights. This appears to support the observation that leptin levels are driven by the lipid content and size of the adipocyte rather than the overall body weight with the LRS group having significantly larger adipocytes [29]. Levels of adiponectin were also significantly higher in the LRS animals, appearing to be at odds with the findings of elevated leptin levels, larger adipocytes and generally higher insulin:glucose ratio observed in these animals. Leptin and adiponectin is known to be inversely regulated *in vivo* [30], however, this relationship is not observed *in vitro* [31], suggesting that the inverse relationship observed *in vivo* is mediated to some extent by indirect mechanisms. Huypens [32] hypothesize the inverse relationship between leptin and adiponecti may operate *via* a hypothalamic mechanism. Evidence for neural regulation include that stimulation of the adrenal-sympathetic axis by leptin results in potent inhibition of adiponectin production and systemic insulin sensitivity [33]. Increased central leptin availability rapidly induces SOCS-3 expression in adipose tissue [34] and markedly lowers circulating adiponectin levels in normal and ob/ob mice [35]. Further, hypertensive patients exhibiting chronic inhibition of the sympathetic drive have increased circulating adiponectin [36]. In our study, the paradoxical increase in adiponectin levels concomitant with increased leptin levels observed in the LRS mice is consistent with induction of leptin resistance in these mice. This is comparable to the selective hypothalamic leptin resistance observed in mice following gold thioglucose treatment [37]. In such animals, increased adiponectin production was detected during the early phase of the obesity syndrome induced by gold thioglucose [32].

Our team has recently demonstrated the utility of MEMRI in understanding changes in appetite regulation in the hypothalamus, showing the sensitivity of this technique to detect predicable changes between fasted and *ad libitum* fed animals in the hypothalamus [15], and changes following hormonal stimulus [14]. We have recently published validation of this techniques against other gold standard methodologies [13]. In our previous studies, increased Mn^2+^ uptake, i.e., overall increased neuronal activation, was observed in the ARC, PVN and VMN brain areas in fasted animals compared to *ad libitum* fasted animals [14]. Administration of oxyntomodulin to fasted animals, led to a reduction in Mn^2+^ uptake in the VMH and PVN areas, resulting in a pattern of Mn^2+^ uptake similar to that observed in *ad libitum* fed animals [14]. In our current study, Mn^2+^ uptake in the VMH and PVN was significantly lower in the HRS animal compared to the LRS animal, reflecting an animal in a more satiated state. MEMRI was also generally lower in the ARC, although not significantly. Thus, using MEMRI we were able to gain an insight into the impact of low and high RS diets on neuronal activation in hypothalamic areas with roles in appetite regulation. These data are even more surprising considering the lower energy intake in the HRS group. The data suggests that there may be appetite regulatory differences between animals exposed to varying amounts of RS with the LRS animals producing MEMRI data closer to fasted animals and the HRS animals to that of sated animals. This is despite the lower energy intake in the HRS diet. It is possible with the demonstrated effect on central appetite regulation, that there may be effects on animal behaviour, which we did not measure in this experiment, such as a decrease in movement that would account of the similar body weight. Initially, we had expected the MEMRI pattern of the HRS group to be comparable to the fasted state since these animals exhibited a greater food intake and lower energy intake, i.e., appear to have increased appetite. However, food intake is regulated by nutrient sensing hypothalamic neurones with glucoprivic conditions eliciting low ATP concentrations in neuropeptide Y/agouti-related protein (AgRP) neurones resulting in induction of AgRP expression and so, increased food intake [38]. Due to the low glycemic nature of a diet in which the carbohydrate component is fulfilled by RS, postprandial glucose levels would be relatively lower in animals on the HRS diet compared to those on the LRS diet, explaining the overall increased food intake observed in the former animals: the increased food intake compensating for the low calorific value of the HRS diet. However, once ATP concentrations in AgRP neurones have been restored, AgRP expression is inhibited, leading to a cessation of feeding. The similarity of the MEMRI pattern between the HRS group and animals in the *ad libitum* fed state suggests that ingestion of significant amounts of RS leads to an animal predominantly in a satiated state.

It is likely the observed effects of the RS starch diet on body composition and appetite regulation are multifactorial. For example, increasing RS reduces energy density of the diet. The influence of energy density of the diet on food intake and adiposity has been already demonstrated in other studies both in animal and human [39]. Increasing RS modifies gastric and intestinal motility and increase distal metabolism. These effects could modify the release of gut hormones involved in the control of food intake [8]. The increased distal gut metabolism leads to an increased production of short chain fatty acids. It is possible that short chain fatty acids have a role in adipose tissue remodelling and hepatic metabolism [40]. Increasing resistant starch also reduces glycemix index which is known to modify insulin/glucose response a possible mechanism for appetite reduction as discussed above [4].

In summary, we have demonstrated that a diet rich in RS has a significant impact on adipose tissue patterning and IHCL deposition. We provide evidence that RS has a significant impact on glucose and insulin metabolism, mediated by major changes in adipocyte morphology and metabolism. MEMRI data suggest that RS can significantly influence hypothalamus areas with a major role in appetite regulation. The MEMRI patterning in the HRS group are similar to those of a satiated animal. This is despite the higher plasma leptin in the lower RS group. This study highlights the need for integrated physiological inquiry to understand the mechanisms behind nutrient induced physiological change. If these observations are reflected in humans, then there is the potential for major health benefits of including RS as the major carbohydrate in the diet, which may support provisional epidemiological evidence of reduced diet related disease in population who consume high levels of RS.

## Materials and Methods

Mice were given diets supplemented with either the RS starch, Hi-Maize™ (National Starch and Chemical Company, Lancashire, UK.) which is 60% RS, or the readily digestible starch, AMIOCA™ (National Starch and Chemical Company, Lancashire, UK.) which contains no RS. We used RS as a model of fermentable dietary fibre. Whole body adiposity, intrahepatocellular lipid (IHCL) and intramyocellular lipid (IMCL) content were measured using ^1^H MRI and MRS methods. MEMRI was also used to measure hypothalamic neuronal activity in the ad libitum fed state at the end of the study period. Body weights and food intake, over the 8 week period of dietary intervention were also recorded. At the end of the interventional period, adipocytes were isolated from epididymal adipose tissue to determine insulin sensitivity and adipocyte size

### Animals and Treatment

All animal studies were performed in accordance with the UK Animals Scientific Procedures Act (1986). Animals were allowed ad libitum access to drinking water and chow. Animals where randomly assigned to either the high or low RS diet (Harlan, Bichester., Oxfordshire, UK) table 2. Animals were housed in groups 4 per cage under conditions of controlled temperature (21–23°C) and light (12-h light, 12 h dark cycle; lights on at 07:00 h). Forty male mice (C57BL/6, 3 weeks old, Harlan UK) were obtained and whole body ^1^H MRS performed (as described below) following a 16 h fast. Based on whole body adiposity measurements by MRS and body weight data, mice were allocated into groups of four and housed in individually ventilated cages.

**Table 2:** Metabolisable energy content of Mice feed

Ingredient	High Resistant Starch			Low Resistant Starch		
	g/kg	kJ/g	kJ/kg	g/kg	kJ/g	kJ
Casein	200	15	2985	200	15	2985
DL-Methionine	3	17	50	3	17	50
Maltodextrin	120	16	1906	120	16	1906
HI-MAIZE 260 (Resistant Starch)*	530	7	3545	0	0	0
AMIOCA TF (Waxy Maize Starch)	0	0	0	530	15	8042
Soybean Oil	50	38	1881	50	38	1881
Cellulose	45	0	0	45	0	0
Mineral Mix	35	3	102	35	3	102
Calcium Phosphate	5	0	0	5	0	0
Vitamin Mix	10	7	71	10	7	71
Choline Bitartrate	3	0	0	3	0	0
TBHQ	0	0	0	0	0	0
Energy density (kJ/gram)			10			15

Energy content estimated from fermentation salvage of resistant starch

Mice were then maintained for 8 weeks on either a low RS diet (LRS, 6 cages of 4 animals) or a high RS diet (HRS, 4 cages of 4 animals). The compositions of the LRS and HRS diets were nutritionally similar ( 69% carbohydrate, 20% protein and 11% fat ) except the former contained AMIOCA™ starch (53% available carbohydrate of feed by weight) as the carbohydrate component whilst the latter contained Hi-Maize™ starch (32% non digestible carbohydrate and 21% available carbohydrate of feed by weight). In calculating the energy content of the feed allowance was made for fermentation and recovery of energy from the RS. This had an impact on the energy density of the feed with AMIOCA™ providing 15kJ/g and Hi-Maize™ providing 10.5 kJ/g (Table 1). This diet was similar to that used by Pawlak et al [7]

Whole body MRI was also performed at weeks 4 and 8 post-dietary intervention as well as *in vivo* localised ^1^H MRS of the liver and muscle at the latter time-point for selected weight-matched animals (n = 8 and 11 for HRS and LRS, respectively). Weekly body weights and food intakes were also recorded throughout the dietary interventional period.

At the end of the interventional period, fasting blood was obtained by cardiac puncture from the animals which had been assessed by whole body MRI and localized ^1^H MRS. Blood was collected into lithium heparin tubes prior to centrifugation for separation of plasma. A sample of epididymal fat (white adipose tissue) was retained for adipocyte isolation (as described below).

### Magnetic Resonance Studies

#### Measurement of Whole Body Adiposity and Individual Adipose Tissue Deposits

Anaesthesia was induced and maintained by inhalation of 1–2% isoflurane/oxygen mix. Following a 16 h fast, whole body ^1^H MRS spectra, to assess whole body adiposity, were obtained on a Varian Inova 4.7T system (Varian Inc., CA, USA) with the following parameters: repetition time (TR) 20 s, 45° pulse and 4 averages. The total percentage adiposity was calculated as previously described [41].

Whole body MRI was also performed at the end of the 8-week dietary interventional period to determine the amounts of internal and subcutaneous fat. A standard spin-echo MRI sequence was employed with TR 2200 ms, echo time (TE) 20 ms, matrix size 256×192, field of view (FOV) 45×45 cm, 2 averages and 50 contiguous slices of 2 mm thickness. Segmentation analysis (SliceOmatic™, Tomovision®, Canada) was performed blindly by the observer on whole body MRI data sets from selected weight-matched animals (n = 8 per dietary group) to determine levels of visceral and subcutaneous adipose tissue.

#### Measurement of Intra-Hepatocellular (IHCL) and Intra–Myocellular (IMCL) Lipid Levels

Localised ^1^H MRS of the liver and muscle was performed at the end of the 8-week interventional period to assess intracellular lipid levels. Placement of a 3×3×3 mm voxel in the organs was guided by pilot MRI images. Using a PRESS sequence with parameters: TR, 10 s; TE, 9 ms and 64 averages, localised ^1^H MRS from the voxels were collected. Relative IHCL and IMCL levels were calculated as previously described [42].

#### Hypothalmic Neuronal Manganese-Enhanced MRI

MEMRI scans were performed on *ad libitum* 4 animals (weight matched) from each dietary group as previously described on a Varian Inova 9.4T scanner (16). Briefly, spin-echo multi-slice T1-weighted imaging was performed with the following scanning parameters: TR 600 ms; TE 10 ms, matrix size = 256×192, FOV 25 mm×25 mm and 1 transient, before and at various times after intravenous (IV) infusion of manganese chloride (62.3 mM, 5 µl/g MnCl_2_.4H_2_O, Sigma-Aldrich, Poole, U. K.). Each scan yielded ten contiguous transverse slices of 1 mm thickness with ∼100 µm resolution, taking a total scan time of 1 min 57 s. The slice offset for each mouse was aligned to identifiable anatomical features (the 4th ventricle and the anterior pituitary gland) with reference to a standard mouse brain atlas [43] to ensure brains were in register for all mice.

### Primary Adipocyte Studies

The isolation of adipocytes from epididymal adipose tissue has been described in detail elsewhere [44] and will only be detailed briefly here. The samples were collected into Dulbecco's Modified Eagle Medium (DMEM, Sigma-Aldrich) containing 5% bovine serum albumin (BSA, Sigma-Aldrich). The epididymal adipose tissue samples were finely minced and DMEM, supplemented with 5% BSA and collagenase (Sigma-Aldrich) were added. The tissue suspensions were placed in a vibrating water bath at 40 cycles/minute (45 mins, 37°C). After incubation, cell suspensions were then filtered through a 400 nm mesh and washed 3 times in a glucose-free Krebs-Ringer phosphate (KRP) buffer with 5% BSA (each wash involving 5 inversions). At no time were the adipocytes left to stand for more than 5 mins without being agitated. *In vitro* studies (see below) were then performed on approximately 30,000 isolated adipocytes. Cells were kept suspended throughout in a vibrating water bath.

#### Measurement of Adipocyte Size

Following adipocyte isolation, an aliquot of the cell suspension was observed by optical microscopy with a graticle placed over one objective lens. The cells were manually counted in a 1:100 dilution of the cell concentrate by means of a haemocytometer (with a 200 µm gap) and the size measured using the graticle scale.

#### Measurement of Adipocyte Insulin-stimulated Glucose Uptake

This has been described in detail elsewhere [21], [44]. The adipocytes were initially incubated (45 minutes, 37°C) in 500 µL of KRP with 5% BSA in the presence of 1 nM insulin (Sigma-Aldrich) before the addition of 300 nM of a non-metabolizable radiolabeled glucose analogue (0.1 µCi C-14 2-deoxy-D-glucose). They were then incubated for a further 15 minutes before centrifugation through 500 µL of silicone oil. Glucose uptake was calculated following liquid scintillation counting of the radiolabeled glucose analogue tracer in the isolated adipocytes.

### Measurement of Plasma Parameters

Plasma from fasted mice was taken for measurement of hormones and selected adipokines using fluorescent bead based assays according to manufacturer's instructions. Leptin, resistin, insulin, total plasminogen activator inhibitor-1 (tPAI-1), interleukin-6 (IL-6), and monocyte chemotactic protein-1 (MCP-1) were measured using a multiplex kit and adiponectin, a single plex kit (Linco Research, St. Charles, MO, USA) on a Luminex system (Austin, Texas, USA). Plasma glucose was measured using a One Touch Ultra meter and test strips (Milpitas, CA, USA).

### Statistical Analysis

Values are quoted as mean±standard error of mean (SEM). Significant differences between measured parameters from the HRS and LRS groups were tested using either the Student's t-test or Mann-Whitney test, except for measurements of percentage adiposity, food intake and body weight which was tested for significance using the two way ANOVA with Bonferonni post-testing. MEMRI data obtained from the two dietary groups was tested for significance by repeated measures ANOVA. Significance was taken as P<0.05
